# Leukemia‐associated Rho guanine‐nucleotide exchange factor is not critical for RhoA regulation, yet is important for platelet activation and thrombosis in mice

**DOI:** 10.1111/jth.13129

**Published:** 2015-10-20

**Authors:** C. M. Williams, M. T. Harper, R. Goggs, T. G. Walsh, S. Offermanns, A. W. Poole

**Affiliations:** ^1^ School of Physiology & PharmacologyUniversity of BristolBristolUK; ^2^ Max Planck Institute for Heart and Lung ResearchBad NauheimGermany; ^3^ Department of PharmacologyUniversity of CambridgeCambridgeCB2 1PDUK; ^4^ Department of Clinical SciencesCornell UniversityIthacaNYUSA

**Keywords:** gene knockout, Larg protein, mouse, mouse, platelets, RhoA protein, mouse, thrombosis

## Abstract

**Background:**

RhoA is an important regulator of platelet responses downstream of Gα_13_, yet we still know little about its regulation in platelets. Leukemia‐associated Rho guanine‐nucleotide exchange factor (GEF [LARG]), a RhoA GEF, is highly expressed in platelets and may constitute a major upstream activator of RhoA. To this end, it is important to determine the role of LARG in platelet function and thrombosis.

**Methods and results:**

Using a platelet‐specific gene knockout, we show that the absence of LARG results in a marked reduction in aggregation and dense‐granule secretion in response to the thromboxane mimetic U46619 and proteinase‐activated receptor 4–activating peptide, AYPGKF, but not to adenosine diphosphate. In a ferric chloride thrombosis model *in vivo*, this translated into a defect, under mild injury conditions. Importantly, agonist‐induced RhoA activation was not affected by the absence of LARG, although basal activity was reduced, suggesting that LARG may play a housekeeper role in regulating constitutive RhoA activity.

**Conclusions:**

LARG plays an important role in platelet function and thrombosis *in vivo*. However, although LARG may have a role in regulating the resting activation state of RhoA, its role in regulating platelet function may principally be through RhoA‐independent pathways, possibly through other Rho family members.

## Introduction

Platelets are critical for the formation of thrombi in blood vessels, leading to rapid occlusion and tissue infarction. An understanding of their function, at a molecular level, is vital to enable a better understanding of the pathophysiology of thrombotic ischemic disease and development of approaches for diagnosis and management. Platelets express multiple members of the Rho family of small GTPases, whose role in many cell types is associated with actin cytoskeletal rearrangements and membrane and granule trafficking 1, 2, 3, 4. In turn, these GTPases are regulated by the balanced activities of Rho activators, the guanine nucleotide exchange factors (GEFs), and Rho inhibitors, the GTPase activating proteins (GAPs). Oligophrenin‐1 has recently been shown to be an important RhoGAP in platelets 5, but the activity of expressed RhoGEFs in platelets is currently sparsely reported.

The most abundantly expressed RhoGEF in platelets is leukemia‐associated RhoGEF (LARG, also known as ARHGEF12) 6, a G protein–coupled receptor (GPCR)‐regulated GEF. Previously, RhoA has been described as an important regulator of platelet function and development, with the generation of a platelet‐specific RhoA knockout showing macrothrombocytopenia, reduced platelet function in response to the thromboxane mimetic, U46619, and thrombin and also reduced thrombus formation *in vivo* 4. Based on the high level of LARG expression in platelets and its specificity for RhoA 7, we hypothesized that LARG would be the principal signal transducer from Gα_13_‐coupled receptors to RhoA 8.

Here, we present data that demonstrate LARG is an important regulator of platelet function and thrombosis *in vivo* but suggest that agonist‐induced RhoA activation does not primarily depend on LARG. As such, the regulation of RhoA may principally be mediated by GEFs other than LARG, and LARG may act independently of RhoA.

## Methods

### Mice

Mice carrying a floxed allele of the gene encoding LARG (*Arhgef12*) were generated as previously described 9 and crossed with PF4‐Cre–positive mice for the generation of platelet‐specific knockouts. Studies were approved by the local research ethics committee at the University of Bristol, UK, with mice bred and maintained under the UK Home Office project license PPL 30/2908.

### Preparation of platelet‐rich plasma

Platelet‐rich plasma (PRP) was prepared from heparinized blood (100 U mL^−1^ 1:10 v/v) taken via cardiac puncture and supplemented with 10 U mL^−1^ heparin (1:5 v/v). Briefly, heparinized blood was spun in a fixed bucket microcentrifuge at 200 × *g* for 5 min. The PRP and top third of the erythrocyte layer were spun again in a swing bucket centrifuge at 200 × *g* for 6 min. PRP was standardized to 2 × 10^8^ platelets mL^−1^ in modified HEPES‐Tyrode's buffer. The residual erythrocyte layer was spun at 1000 × *g* in a fixed bucket microcentrifuge. PPP was removed and diluted to the same extent as the appropriate PRP.

### Washed platelet preparation

Washed platelets were prepared from citrated blood (4% sodium citrate, 1:10 v/v) taken via cardiac puncture and supplemented with acid‐citrate dextrose (1:7 v/v). Platelets were pelleted from PRP in the presence of 140 nmol L^−1^ prostaglandin E_1_ and 0.02 unit mL^−1^ apyrase. The platelet pellet was the resuspended at a concentration of 2 × 10^8^ platelets mL^−1^ in modified HEPES‐Tyrode's buffer containing 0.02 unit mL^−1^ apyrase. For certain assays, 10 μmol L^−1^ indomethacin was added before and after the pelleting stage.

### Western blotting

Western blotting samples were prepared from washed platelet preparations. Rabbit anti‐LARG (clone H70) and rabbit anti‐RhoA (clone 26C4) were from Insight Biotechnology (Wembley, UK). Mouse anti–α‐tubulin was from Sigma UK (Poole, UK).

### Flow cytometry

Washed platelets at 2 × 10^7^ platelets mL^−1^ were incubated with 1/25 dilution from stock of FITC‐conjugated rat anti‐mouse antibodies against CD41/α_IIb_, glycoprotein (GP)VI, and GPIbα or isotype‐specific controls, for 10 min at room temperature, before fixation with an equal volume of 4% paraformaldehyde. Samples were analyzed on a BD FACS Canto II flow cytometer (BD Biosciences, Oxford, UK) with FACS Diva software.

### Lumiaggregometry

Lumiaggregometry was performed on a ChronoLog Corp. Model 560‐VS aggregometer and Aggrolink 5 software (Lab Medics, Abingdon On Thames, UK). Manufacturer's instructions were followed, with the exception that the aggregations were scaled down to 250 μL. Maximum extent and rate of aggregation were calculated using the Aggrolink 5 software.

### Thromboxane B_2_ ELISA

Releasates were generated under aggregating conditions before being quenched with 5 mmol L^−1^ EDTA and 200 μmol L^−1^ indomethacin. Releasates were centrifuged for 3 min at 10 000 × *g*, decanted, and snap frozen. Releasates were diluted 1:50 before analysis of thromboxane B_2_ levels via a commercial ELISA kit (Enzo Life Sciences, Exeter, UK) according to the manufacturer's instructions as previously described 10.

### RhoA G‐LISA

Washed platelets were stimulated under non‐stirring conditions before the addition of an equal volume of ice‐cold 2× lysis buffer (100 mmol L^−1^ Tris‐Cl, 1 mol L^−1^ NaCl, 10 mmol L^−1^ MgCl_2_, 2% Triton X‐100) containing Compete, EDTA‐free protease inhibitors (Roche Diagnostics Ltd, Burgess Hill, UK). Lysates were analyzed via commercial RhoA G‐LISA kit (Cytoskeleton Inc., Universal Biologicals, Cambridge, UK) according to the manufacturer's instructions.

### Spreading assays

Platelets containing indomethacin at 4 × 10^7^ platelets mL^−1^ were added onto coverslips coated in 50 μg mL^−1^ collagen‐related peptide or 100 μg mL^−1^ fibrinogen, or BSA as a control, for 30 min at room temperature. Cells were fixed with an equal volume of 8% paraformaldehyde, washed twice with PBS, and stained with 2 μmol L^−1^ DiOC_6_ for 1 h at room temperature. Platelets were washed twice before being mounted with MOWIOL solution containing DABCO. Platelets were imaged on a Leica DM IRM epifluorescence microscope with five random fields of view captured using Volocity Image Analysis software (Perkin Elmer, Coventry, UK). Images were analyzed using ImageJ 1.46.

### *In vivo* thrombosis assays

Mice were anesthetized with 100 mg kg^−1^ ketamine (Vetalar V, Pfizer) and 10 mg kg^−1^ xylazine (Rompun, Bayer). Platelets were labeled by intravenous administration of 100 mg kg^−1^ Dylight 488–conjugated anti‐GP_Ibβ_ antibody (Emfret Analytics, Eibelstadt, Germany). Right arteries were exposed, and 2 × 1‐mm pads of filter paper soaked with ferric chloride in PBS were applied for 3 min. Injury sites were imaged by time‐lapse microscopy for 20 min, and images were analyzed by ImageJ 1.46. Background fluorescence values measured upstream of the injury site were subtracted from the thrombus‐specific fluorescence. Data are expressed as integrated fluorescence density.

### *In vitro* thrombosis assays

*In vitro* thrombosis assays were performed on a parallel‐plate flow chamber. Coverslips were coated with 50 μg mL^−1^ HORM collagen for 2 h at room temperature before being blocked overnight at 4 °C with 1% fatty acid–free BSA (Sigma, Poole, UK). Assembled chambers were blocked for 30 min at room temperature before a run. Blood was taken into 4% citrate (1:10 v/v) supplemented with 2 units mL^−1^ heparin and 40 μmol L^−1^ PPACK. Blood was incubated with 2 μmol L^−1^ DiOC_6_ for 10 min at room temperature. Blood was allowed to flow at 1000 s^−1^ for 2 min. Images were captured by time‐lapse microscopy, with five randomly selected fields of view captured for end‐point analysis. Images were analyzed by ImageJ 1.46.

## Results and discussion

**Figure 1:**
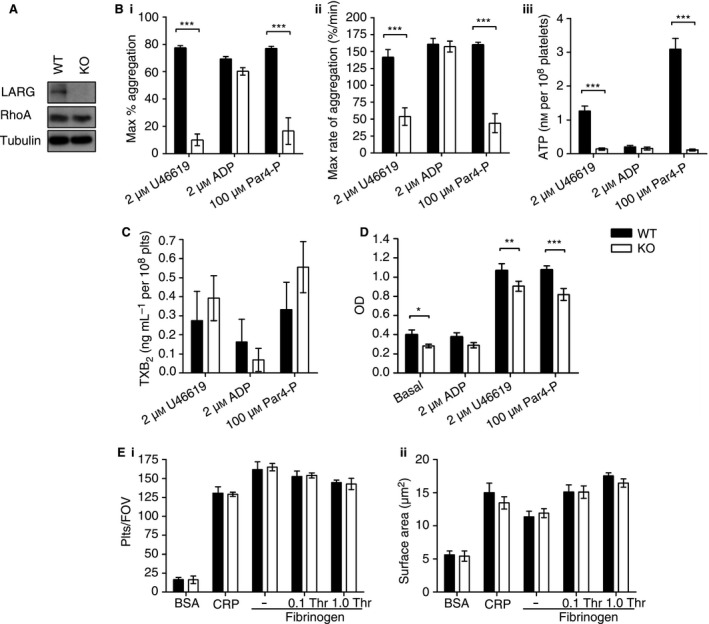
Leukemia‐associated Rho guanine‐nucleotide exchange factor (LARG)‐null platelets are developmentally normal, but demonstrate reduced activity to the thromboxane mimetic U46619 and proteinase‐activated receptor 4 (PAR4) peptide AYPGKF. (A) Platelet lysates from wild‐type (WT) and knockout (KO) mice were probed via Western blotting (*n* = 3). (B) Platelet‐rich plasma (PRP) from WT and KO mice was analyzed via lumiaggregometry to determine (i) the maximum extent of aggregation within 3 min, (ii) the maximum rate of aggregation, and (iii) the amount of adenosine triphosphate (ATP) secretion as a readout of dense‐granule secretion (*n* ≥ 3). Data were compared by 2‐way anova with ***P <* *0.001. (C) PRP from WT and KO mice was stimulated under stirring conditions for 5 min. Reactions were stopped with 5 mmol L^−1^ EDTA and 200 μmol L^−1^ indomethacin. Releasates were harvested via centrifugation before being analyzed for thromboxane (TX)B_2_ levels by ELISA (*n* ≥ 3). (D) RhoA activity was measured by G‐LISA. Washed platelets from WT and KO mice at 2 × 10^8^ platelets mL ^−1^, supplemented with 0.02 unit mL ^−1^ apyrase, were stimulated under non‐stirred conditions for 5 min at 37 °C (*n* = 7). Data are shown as optical density (OD) and were compared by 2‐way anova with *P <* *0.05, **P <* *0.01, and ***P <* *0.001. (E) Washed platelets were applied to BSA, collagen‐related peptide (CRP), or fibrinogen‐coated surfaces for 1 h before fixing and staining. Adhesion was determined as being the number of adhered platelets per field of view (i), and platelet spread area was determined by fluorescence (ii). Platelets applied to fibrinogen‐coated surfaces were co‐stimulated with either 0.1 unit mL ^−1^ or 1.0 unit mL ^−1^ thrombin to stimulate spreading on that surface.

Platelets from LARG:PF4‐Cre^+^ (knockout) mice were confirmed to be deficient in LARG, yet still expressed wild‐type levels of RhoA (Fig. 1A). Hematologic analysis of knockout mice showed that absence of LARG from platelets did not impair hematopoiesis. Likewise, the absence of LARG in platelets did not affect the basal cell surface expression of key platelet GPα_IIb_, GPIb, and GPVI (Table 1). This is unlike the RhoA knockout where a macrothrombocytopenia was present 4, indicating that the absence of LARG, unlike RhoA, does not impact the development and generation of platelets *in vivo*.

**Table 1:** Hematology and platelet surface receptor analysis

	WT			KO		
	Mean	SEM	*n*	Mean	SEM	*n*
Hematology						
WBC* (10^3^ mm^−3^)	7.26	0.70	11	5.73	0.70	11
RBC† (10^6^ mm^−3^)	10.13	0.19	11	10.18	0.24	11
PLT‡ (10^3^ mm^−3^)	749.6	47.2	11	804.0	56.9	11
MPV§ (μm^3^)	5.32	0.15	11	5.26	0.06	11
Surface receptors						
GPα_IIb_	3127	115	4	3139	212	5
GPIb	713.8	53.6	4	754.5	49.2	5
GPVI	320.9	9.7	4	325.8	11.7	5

Hematology data of citrated whole blood were acquired using a Horiba Pentra ES60. Platelet surface receptors were determined by flow cytometry. Washed platelets from wild‐type (WT) and knockout (KO) mice were incubated with fluorescently conjugated antibodies against GPα_IIb_, GPIb, and GPVI and then fixed. Data are presented as geometric means. *White blood cells. ^†^Red blood cells. ^‡^Platelets. ^§^Mean platelet volume.

Aggregation of knockout platelets in PRP was diminished in response to the thromboxane mimetic U46619 and the protease‐activated receptor 4 (PAR4) activating‐peptide (PAR4‐P) AYPGKF but not to adenosine diphosphate. Dense granule secretion, as measured by adenosine triphosphate release in a luciferin‐luciferase assay (Fig. 1B), was also reduced in knockout platelets; however, thromboxane generation was not (Fig. 1C). The defect in thromboxane and PAR4‐dependent responses and not those to adenosine diphosphate suggests that LARG is important in Gα_12/13_‐linked GPCR responses and not those linked to Gα_i_/Gα_q_. Adhesion and spreading of LARG‐deficient platelets on fibrinogen or collagen‐related peptide (CRP) under static conditions were also normal (Fig. 1E), suggesting that LARG does not act downstream of α_IIb_β_3_ integrin–associated Gα_13_^11^.

Importantly, RhoA activity was reduced in knockout platelets compared with wild‐types in both resting and agonist‐stimulated platelets, when measured with G‐LISA (Fig. 1D). When normalized to basal activity (not shown), there was no longer a difference in stimulated RhoA activity between controls and LARG‐deficient platelets, suggesting that LARG regulates basal RhoA activity but is not critical for activating RhoA after platelet stimulation. LARG was a good candidate to be the primary GEF regulating RhoA activity in platelets, so it is therefore surprising that RhoA activity was not altered in an agonist‐dependent manner. It could be that LARG plays a ‘housekeeping’ role, maintaining a background level of RhoA activation, such that its absence results in a cell that is less responsive, and that RhoA agonist‐dependent activation occurs through other RhoGEFs. The uncoupling of the apparent functional roles of LARG from RhoA activation may also be due to LARG regulating another Rho family member in platelets 12. However, given that both RhoF 2 and RhoG 1, 13 knockout mice present dissimilar phenotypes, these Rho family members are unlikely to be regulated by LARG, suggesting that RhoB or RhoC may be involved, despite their low copy numbers 14. It has also been reported that LARG can form homodimers or heterodimers with PDZ‐RhoGEF, via interactions with their inhibitory C‐terminal domains that may recruit other inhibitory factors 15. Although PDZ‐RhoGEF is not reported to be expressed in platelets at the protein level 14, the suggestion that dimerization may recruit other inhibitory factors could be important for explaining the regulatory role of LARG in platelets.

**Figure 2:**
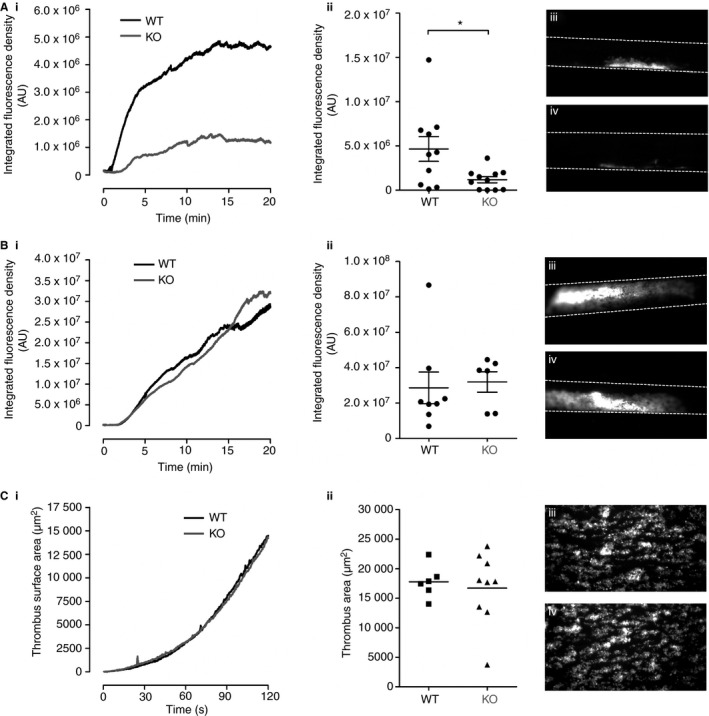
Mice with leukemia‐associated Rho guanine‐nucleotide exchange factor (LARG)‐null platelets demonstrate reduced thrombus formation in response to low‐level injury *in vivo*. Exposed carotid arteries from wild‐type (WT) and knockout (KO) mice were injured with (A) 6% (WT *n* = 10, KO *n* = 11) or (B) 12% ferric chloride (WT *n* = 8, KO *n* = 6) for 3 min. Thrombus formation was monitored for 20 min. Data were analyzed using ImageJ 1.46, yielding (i) the mean integrated fluorescence density over time and (ii) the thrombus size after 20 min. Representative images of thrombi from (iii) WT and (iv) KO. Data were compared by unpaired *t*‐test where appropriate. *P <* *0.05. (C) Whole blood was flowed through a parallel‐plate flow chamber over a collagen‐coated surface. Platelet accumulation was followed for 2 min by time‐lapse microscopy (i) with still images taken for end point analysis (ii). Representative images of thrombi from (iii) WT and (iv) KO.

Analysis of thrombus formation *in vivo* revealed knockout mice were also protected from thrombosis induced at lower (6%) concentrations of FeCl_3_ (Fig. 2A), although at higher concentrations (12%) of FeCl_3_, this protection was overcome (Fig. 2B). Likewise, thrombus formation on collagen‐coated surface *in vitro*, under non‐coagulating conditions, showed no difference between wild‐type and knockout mice (Fig. 2C). The presence of an *in vivo* defect under milder injury conditions is consistent with a defect in thrombin‐dependent thrombus formation 16. The absence of a difference in thrombus formation under non‐coagulating conditions *in vitro*, where thrombin is not generated, is also consistent with a Gα_12/13_‐link.

We conclude that the Rho‐GEF LARG has an important role in regulating platelet aggregation downstream of Gα_13_‐coupled receptors (thrombin and thromboxane receptors). Importantly, this translates into a defect in arterial thrombosis *in vivo*. Although we had hypothesized that LARG may be operating through RhoA to mediate these effects, the difference in phenotypes between LARG‐deficient and RhoA‐deficient platelets and our discovery here that only basal RhoA activity is affected by absence of LARG points to other pathways that LARG may regulate to mediate its effects. Given the marked effect on dense granule secretion, we suggest that LARG may be operating as a novel regulator of secretion downstream of Gα_13_‐coupled receptors.

## Addendum

C. M. Williams designed and performed experiments, analyzed data, and wrote the manuscript. M. T. Harper, R. Goggs, and T. G. Walsh designed and performed experiments, analyzed data, and edited the manuscript. S. Offermanns provided essential reagents and edited the manuscript. A. W. Poole designed experiments and wrote the manuscript.

## Disclosure of Conflict of Interests

The authors state that they have no conflict of interest.
